# The assessment of the amount of water pollution and its suitability for drinking of the Tyśmienica River Basin, Poland

**DOI:** 10.1007/s10661-021-09034-w

**Published:** 2021-04-30

**Authors:** Antoni Grzywna, Joanna Sender

**Affiliations:** University of Life Sciences in Lublin, Lublin, Poland

**Keywords:** Water quality, Water pollution index, Drinking purpose, Tyśmienica River Basin

## Abstract

The quality and potability of waters of the Tyśmienica River Basin were determined in 2017 at eight measuring stations. The paper presents analyses of the physical and chemical parameters of surface waters of the basin. With regard to suspended solids, pH value, electric conductivity, sulphates, ammonia, chlorides and phosphate, the water was classified as having a very good ecological status. In the case of Kjeldahl nitrogen, the waters were classified as having a good ecological status. In the case of the remaining parameters, i.e. BOD, DO, TOC and COD, the status varied among stations. The values of all the physical and chemical parameters complied with the values for undisturbed conditions. Only ammonia and COD showed significant differences among stations. The WPI values for each measuring point ranged from 0.90 to 1.56, what corresponds to the descriptive indicators of moderately polluted water. The high concentrations of COD and TOC indicate that high-performance treatment processes (category A3) must be used to ensure that the water is suitable for drinking.

## Introduction

According to the EU Water Framework Directive, member states are required to guarantee and maintain a good ecological status of surface and ground waters (WFD, 2000). The key parameters include total phosphorus, dissolved phosphorus, ammonia, total nitrogen, organic nitrogen and nitrate nitrogen contents. Human activity in the river basin is the key determinant of surface water quality. Among the major factors influencing the aquatic environment are significant intake of water for household and economic use, discharge of insufficiently treated effluents and wastewater and agricultural run-off contaminated with nutrients (Brankov et al., 2012; Mouri et al., 2012; Kanownik et al., 2013; Lai et al., 2013).

Nitrogen and phosphorus compounds from point and diffuse sources pose special hazard to the aquatic environment. They give rise to the risk of enriching the aquatic environment with nutrients, and as a result, the eutrophication of water bodies would happen. In the process of eutrophication, the productivity of water is enhanced by increasing the content of nutrients in water (Sobczyński & Joniak, 2013; Kowalik et al., 2014; Neverova-Dziopak & Preisner, 2015). Excessive input of such substances leads to changes in the species structure of the flora and fauna of the aquatic environment, and in consequence, deterioration of the ecological and chemical status of rivers. The accumulation of large quantities of algae inhibits photosynthesis and stimulates toxic effects of the metabolites and decomposition products of the necrotic algae and causes deterioration of oxygen conditions. Eutrophication has become a global issue. It can be observed in seas and rivers as well as lakes all over the world. Eutrophication constitutes the most frequent anthropogenic disturbance to the functioning of water ecosystems which causes the most significant burdens (Grzywna et al., 2015; Sojka et al., 2016)

The influx of nutrients from the basin is a result of both natural processes taking place in the basin and anthropogenic factors. Natural processes having an impact on surface water quality are climate and hydrological conditions, topography, geological structure, soil type and soil erosion (Policht-Latawiec et al., 2014; Liberacki & Szafrański, 2008; Grzywna & Kowalczyk-Juśko, 2018; Sojka & Murat-Błażejewska, 2009; Varol et al., 2012). Anthropogenic factors include urbanization, industrial and agricultural activity, use and management of land and intensified decrease in water resources (Feher et al., 2016; Grzywna et al., 2017).

Surface and ground waters constitute one of the most essential water sources for humans. Due to various factors, they are the most susceptible to pollution. Above all, they are highly exposed to the risk of pollution from anthropogenic, solid point sources, such as discharges of industrial and municipal sewage as well as agricultural sewage (Sargaonkar & Deshpande, 2003; Alam et al., 2007). Surface runoff, on the other hand, is a seasonal phenomenon; spatial and temporal variability of rainfall, interflow and groundwater flow have the greatest importance (Liao et al., 2008; Ji et al., 2013; Takić et al., 2012).

Ouyang et al. (2006) point out to the significance of controlling and monitoring the quality of water in agricultural catchments. Necessary measures include a monitoring program, evaluating the sources of pollution, investigating the environmental conditions on the site and assessing the ecosystem services in a reliable manner. The quality of surface water and ground water in the basin is determined by its chemical composition. Therefore, the physicochemical properties of water were tested in order to assess its ecosystem services (Nouri et al., 2008; Khadka & Khanal, 2008; Iticescu et al., 2013; Seth et al., 2015; Spiess, 2011)

Both individual and cumulative ratios can be used in evaluating the quality of water. Nevertheless, it is not easy to evaluate the quality of water for multiple samples based on more than one parameter. To ensure a specific assessment, attempts were made to create a mathematical index model. The Water Quality Index was developed for the first time in the USA (Horton, 1965). In the following years, many water quality indices used in evaluation of overall water quality were developed throughout the world. These indicators varied in the way they were calculated and used in the management of water resources. The most popular indices included National Sanitation Foundation Water Quality Index (NSFWQI), Canadian Council of Ministers of the Environment Water Quality Index (CCMEWQI) and British Columbia Water Quality Index (BCWQI). They were developed for a specific region only (Alam et al., 2007). Due to the need for global acceptance, a uniform Weighted Arithmetic Water Quality Index method (WAWQI) was developed. It assigns weights and determines the arithmetic mean using the most frequently measured variables describing water quality. However, the choice of parameters and weights assigned to them are often subjective (Tyagi et al., 2013; Misaghi et al., 2017). The need to find a useful universal water quality indicator resulted in the development of the Water Pollution Index based solely on comparing water quality parameters with regulatory standards. Its basic advantage is the simplicity of calculation. This paper aims to assess the actual ecological status of the surface water in the Tyśmienica River Basin in 2017, its water pollution index and suitability for drinking. It is important that a monitoring program is developed to observe deterioration in surface water quality. Such monitoring facilitates an evaluation of the sources of pollution and helps understand the environmental conditions on the site and ensure proper management of water resources (Khadka & Khanal, 2008; Seth et al., 2015).

The quality of water is frequently misinterpreted as a final ecosystem service. As a matter of fact, it makes an important contribution to the emergence of many different services, from recreation to human health (Keeler et al., 2012). For surface water, it will be irrigation water supply, natural purification of water; erosion control; habitat for fish and wildlife; dilution of wastewater; and recreation use (Loomis et al., 2000). In Poland, the surface water is conventionally used for irrigation, drinking, as a habitat for fish as well as for recreation. It should be noted that the requirements of water used for irrigation and for recreation are much lower than for drinking water. The suitability of water resources for human consumption can be assessed using quality parameters. Clean potable water supplied to households is the end product of ecosystem services. The availability of reliable and safe water resources for drinking purposes is essential for a sustainable ecosystem.

## Materials and methods

The River Tyśmienica with an average outflow of 8.6 m^3^ s^−1^ constitutes a 3rd order catchment basin flowing through the West Polesie and South Podlasie Lowland, regions in Poland. The catchment comprises 33 lakes, 3 storage reservoirs and 8 pond complexes. The River Tyśmienica is 74.15 km long and has a basin area of 2750.04 km^2^. It is the largest right-bank tributary of the River Wieprz (at 71.94 km). It is assumed to originate in the drainage ditches of Lake Rogóźno. It is difficult to accurately identify the boundaries of the catchment area due to the presence of peat land and canals connecting the area to the neighbouring catchment basins. The Tyśmienica is a small river in a predominantly peat land valley, for which applicable regulations define the target concentrations of physical and chemical parameters corresponding to the respective class of the ecological status of water. The river basin has many natural assets as it covers numerous protected areas, including the Natura 2000 areas. In addition, it is situated within the West Polesie Biosphere Reserve (Grzywna & Nieścioruk, 2016; Chmielewski et al., 2014).Czarnecka, 2005; Michalczyk & Wilgat, 1999)

**Fig. 1:**
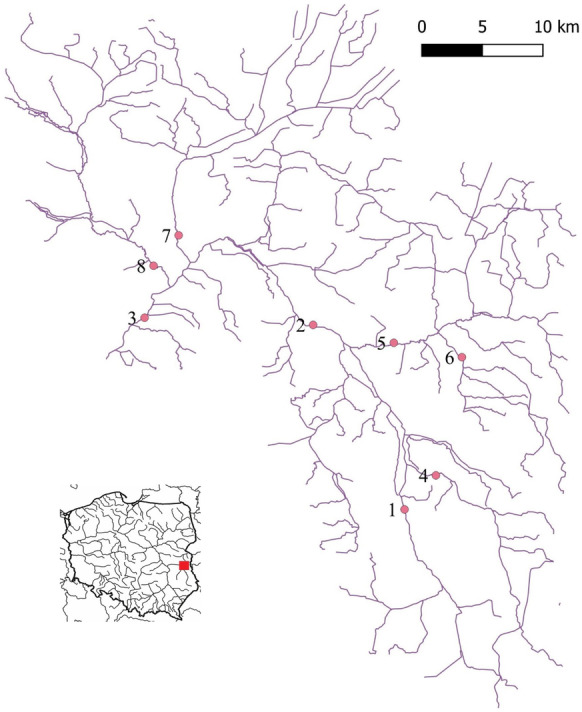
The hydrographic network of the Tyśmienica River Basin

This study was conducted at eight measuring stations—checkpoints (Fig. 1). For water quality assessments, four samples were collected at each station during the year 2017. Table 1 shows the location of the stations on five rivers in the Tyśmienica basin, with specifications of distance to the mouth, discharge and GPS coordinates.

**Table 1:** Location of the stations in the Tyśmienica river basin

L. p.	Station	River	Distance of the mouth [km]	Discharge [m^3^ s^−1^]	Longitude E	Latitude N
1	Buradów	Tyśmienica	53	8	22°27′25″	51°37′50″
2	Niewęgłosz		30	5	22°41′49″	51°41′49″
3	Kock		5	2	22°50′52″	51°31′48″
4	Rudka	Bobrówka	8	1.5	22°53′01″	51°32′10″
5	Parczew	Piwonia	9	1.7	22°54′28″	51°38′51″
6	Uhnin		27	1	23°04′49″	51°35′26″
7	Borki	Bystrzyca	5	2.2	22°31′22″	51°43′29″
8	Białka	Biała	7	1.1	22°35′20″	51°44′20″

In situ recorded pH, dissolved oxygen and electric conductivity (EC) values were measured with a portable multi parameter instrument. The remaining parameters were analysed in the laboratory using standard methods. The contents of phosphates (PO_4_), sulphates (SO_4_), chloride (Cl), ammonium ion (NH_4_), Kjeldahl nitrogen (KN) and total organic carbon (TOC) were determined in the water samples using a PC AQUALYTIC spectrophotometer. Biochemical oxygen demand (BOD) was determined by the Winkler method, chemical oxygen demand (COD) by the bichromate method and suspended solids (SS) by the gravimetric method (Policht-Latawiec et al., 2014; Grzywna et al., 2015; PN–EN ISO 5667–3, 2005).

For each water quality parameter, the average value over the test period was determined. Moreover, the physicochemical indices were subjected to a detailed statistical analysis. First, the normality of the distributions was evaluated using the Shapiro-Wilk test. Then, the significance of differences between the values of the individual parameters was estimated with the non-parametric Kruskal-Wallis test (at the significance level *α* = 0.05). The statistical analyses were performed using Statistica 12PL software.

The ecological status of water in the river at every measurement site was evaluated according to the “Regulation of the Minister of Environment on the method of classification of the state of surface water bodies and environmental quality standards for priority substances” (Gazette, 2016). The ecosystem services of water were evaluated pursuant to the “Regulation of the Minister of the Environment on the requirements to be met by surface waters used to supply the population with drinking water” (Gazette, 2002).

The proposed water pollution index (WPI) method has been described in reference literature (Filatov et al., 2005). The WPI represents an arithmetical way of integrating parameters for assessing the chemical and ecological status of surface waters. Mean WPIs were calculated for the observation periods based on a comparison between mean annual listed parameters and the defined standard values of the chemical parameters of water (Table 2), divided by the number of used parameters (Takić et al., 2017; WHO, 2017). The WPI is calculated based on a set of values for water quality parameters, using the formula:

**Table 2:** The standard threshold values (*T*) for class I and standard drinking water (S)

Parameter	Unit	Value *T*	Value *S*
SS	mg dm^−3^	24.5	25
BOD	mg O_2_ dm^−3^	3.0	5.0
DO	mg O_2_ dm^−3^	7.5	5.0
TOC	mg C dm^−3^	10	-
pH	-	7.0–8.0	7.5
EC	µS cm^−1^	549	1500
SO_4_	mg SO_4_ dm^−3^	42	120
Cl	mg Cl dm^−3^	26	50
NH_4_	mg NH_4_ dm^−3^	0.75	0.8
KN	mg N dm^−3^	1.0	-
PO_4_	mg PO_4_ dm^−3^	0.4	0.4
COD	mg O_2_ dm^−3^	25	25

$$WPI \, = \frac{1}{n}\sum\limits_{n = 1}^{n} {\frac{{A_{{\text{n}}} }}{T}}$$

where *A*_n_ is the annual average value for each parameter, *T* is the standard threshold values for each parameter, *n* is the number of used parameters

Based on the obtained WPI values, watercourses are classified into different classes of quality. If the value of the WPI < 1, the watercourse is marked as pure, if the WPI > 2, the watercourse is polluted, and if WPI > 6, the watercourse belongs to a group of heavily impure waters (Table 3) (Lyulko et al., 2001). In our study, water pollution was estimated and changes in the selected water quality indices for the river along its course were determined using the WPI. In the evaluation of surface water quality, the WPI could be an efficient measure for ecological status assessment, as evidenced by research carried out, for example, in the Potok Goławiecki, the River Danube, the River Timok, the River Sava or a Mediterranean island (Jabłońska, 2008; Takić et al., 2012; Brankov et al., 2012; Iticescu et al., 2013; Popović et al., 2016; Pavlidis et al., 2018).

**Table 3:** Water purity classification based on the WPI value (Takić et al., 2017)

Class	I	II	III	IV	V	VI
Water purity	Very pure	Pure	Moderately polluted	Polluted	Impure	Heavily impure
WPI value	< 0.31	0.31–1.00	1.01–2.00	2.01–4.00	4.01–6.00	> 6.00

The clarity of water and its suitability for consumption by humans can be determined according to global or regional standards. Table 4 presents the limit values for clean water in Poland (Gazette, 2002).

**Table 4:** The limit values of water clarity

Parameter	Unit	A1	A2	A3
SS	mg dm^−3^	25	30	35
BOD	mg O_2_ dm^−3^	3	5	7
DO	mg O_2_ dm^−3^	7	5	3
TOC	mg C dm^−3^	5	10	15
pH	-	6.5–8.5	6.0–9.0	5.5–9.0
EC	µS cm^−1^	1000	1000	1000
SO_4_	mg SO_4_ dm^−3^	150	200	250
Cl	mg Cl dm^−3^	200	200	200
NH_4_	mg NH_4_ dm^−3^	0.5	1.5	2.0
KN	mg N dm^-3^	1	2	3
PO_4_	mg PO_4_ dm^−3^	0.4	0.7	0.7
COD	mg O_2_ dm^−3^	25	30	35

A1 is the water requiring simple physical treatment; A2 is the water requiring typical physical and chemical treatment; A3 is the water requiring highly effective physical and chemical treatment

## Results

As required by the Water Framework Directive, all surface waters must have a good ecological status, i.e. the level of changes resulting from human activity must be kept low (WFD, 2000). In the current study, the ecological status and the water pollution index were determined based on the analysis of physicochemical parameters of water in the selected stations at the Tyśmienica river basin, and the pollutant index was assessed in the context of provision of ecosystem services. The average annual values of physicochemical parameters at stations of the river basin are given in Table 5.

In Table 5, the values of EC, suspended solids, sulphates and chlorides were very low in comparison to the values acceptable for waters with first class quality. Based on the extreme values of pH ranging between 6.9 and 7.9, the tested waters can be considered slightly alkaline. Statistical analysis showed that no significant differences were recorded between selected stations.

Concentrations of DO in the water of the River Tyśmienica ranged from 6.2 to 8.5 mg O_2_ dm^−3^. At all the stations where the values of the DO were greater than 7.0 mg O_2_ dm^−3^, they met the requirements for first class quality (Table 5). On the other hand, the water quality at Koczergi was included in class II. The mean values of BOD at all stations were classified as class I, while the mean value of BOD at Borki was classified as class II. The mean values of TOC and COD at all stations were classified as class II, while the mean value of both parameters at Kock was classified as class I. Statistical analysis showed that no significant differences had been recorded between selected stations.

**Table 5:** Average values and classification of chemical parameters

Indicator	Unit	Kock	Niewęgłosz	Buradów	Borki	Parczew	Białka	Rudka	Koczergi
SS	mg L^−1^	12.3 (I)	14.4 (I)	12.7 (I)	21.2 (I)	15.4 (I)	18.0 (I)	15.0 (I)	18.7 (I)
BOD	mg L^−1^	2.4 (I)	2.2 (I)	2.0 (I)	3.2 (II)	2.1 (I)	1.9 (I)	2.5 (I)	2.7 (I)
DO	mg L^−1^	8.4 (I)	8.0 (I)	8.3 (I)	7.7 (I)	8.0 (I)	8.5 (I)	7.5 (I)	6.2 (II)
TOC	mg L^−1^	8 (I)	11 (II)	12 (II)	13 (II)	13 (II)	11 (II)	14 (II)	15 (II)
pH		7.1 (I)	7.3 (I)	7.7 (I)	7.4 (I)	6.9 (I)	7.7 (I)	7.9 (I)	7.3 (I)
EC	µS cm^−1^	388 (I)	373 (I)	349 (I)	546 (I)	351 (I)	421 (I)	477 (I)	333 (I)
SO_4_	mg L^−1^	26 (I)	17 (I)	22 (I)	41 (I)	22 (I)	16 (I)	25 (I)	28 (I)
Cl	mg L^−1^	7 (I)	11 (I)	20 (I)	22 (I)	13 (I)	15 (I)	17 (I)	25 (I)
NH_4_	mg L^−1^	0.17 (I)	0.21 (I)	0.27 (I)	0.54 (I)	0.22 (I)	0.40 (I)	0.30 (I)	0.44 (I)
KN	mg L^−1^	1.73 (II)	1.63 (II)	1.60 (II)	1.73 (II)	1.91 (II)	1.40 (II)	1.80 (II)	1.86 (II)
PO_4_	mg L^−1^	0.26 (I)	0.33 (I)	0.30 (I)	0.22 (I)	0.23 (I)	0.22 (I)	0.29 (I)	0.20 (I)
COD	mg L^−1^	23 (I)	29 (II)	32 (II)	28 (II)	27 (II)	26 (II)	30 (II)	33 (II)
Ecological class^a^		II	II	II	II	II	II	II	II
WPI		0.90	1.10	1.18	1.56	1.08	0.98	1.22	1.35
WPI class^b^		II	III	III	III	III	II	III	III

^a^I is the first class quality, II is the second class quality

^b^II is the pure, III is the moderately polluted

The nutrient compounds are important parameters of the assessment. Despite a significance differences occurring between the stations, the mean values of phosphates and ammonia did not exceed the threshold for first class quality. The Kjeldahle nitrogen was recorded as the worst parameter, where its mean concentrations ranged from 1.40 to 1.91 mg N dm^−3^. At all the sites, the waters were assigned to second class quality, i.e. good ecological status. Among the analyzed nutrients, only ammonia revealed significant differences between the respective stations.

In case of the salinity parameters, which include electric conductivity (EC), total dissolved solids (TDS), sulphates (SO_4_) and chlorides (Cl), all analyzed water samples were included in the first class. At the Kock station, only one parameter (KN) proved decisive for the second class quality, while at the stations Borki and Koczergi, there were four parameters that are classified as class II. Lower water quality was mainly determined due to the high content of Kjeldahl nitrogen, TOC and COD (Sojka et al., 2016; Popović et al., 2016). The results of the analysis showed that water quality is corresponding to good ecological status at all checkpoints. It can be concluded that the concentrations of pollution do not exceed the range for a good ecological status of water.

Based on the calculated WPI values, it was found that the surface water in the Tyśmienica river basin was in the range of 0.90 to 1.56, which corresponds to pure and moderately polluted water. The results indicated that the lowest WPI values were recorded at the Kock and Białka, where the water quality was classified as pure (WPI < 1.0). At other stations, the water was classified as moderately polluted, and the highest WPI values were recorded at the Borki. The average annual value of WPI at Tyśmienica basin in 2017 was 1.2, which is a characteristic of moderately polluted surface water. The most important parameters with values much higher than the standard values of Class I were TOC, KN, COD. TOC and COD represent the organic pollution, while KN are the main nutrients that affect the eutrophication of aquatic ecosystems (Grzywna et al., 2018). Moderate pollution of water at Tyśmienica basin may result from the mineralization process of peatland. The drying process of peatland contributes to the discharge of nutrients, which are permanent sources of pollution.

One of the most important aspects of water resource management is the determining water availability indicators. The highest requirements for accessibility are associated with the use of clean drinking water. The results of this study showed that the water from all the stations in the Tyśmienica river basin can be supplied for human consumption. Of course, it must be subjected to treatment processes. The water clarity aspect can be classified into 3 categories namely A1, A2 and A3. In the case of clarity category A1, the water requires simple physical treatment, in particular filtration and disinfection. According to applicable regulations, drinking water at all the stations falls into category A1 with respect to pH value, sulphates (SO_4_) and chlorides (Cl), electric conductivity (EC) and total dissolved solids (TDS). With regard to BOD, DO, KN and PO_4_, the river water should be subjected to physical and chemical treatment typically of A2 clarity category. In addition, the water should subject to oxidation, coagulation, flocculation, decanting, filtration and disinfection. Due to high concentrations of TOC and COD, the utilities can be deteriorated, which mean that the water should be subjected to intensive physical and chemical treatment typically of category A3. Then, the water undergoes oxidation, coagulation, flocculation, decantation, filtration, activated carbon adsorption and disinfection. The results of this study indicated hat the Kock was the best station of water supplying for human consumption, which belong to A2 category. On the other hand, the water at other stations belonging to A3 category (Table 6). Clarity classification of water supplied to the population.

## Discussion

**Fig. 2:**
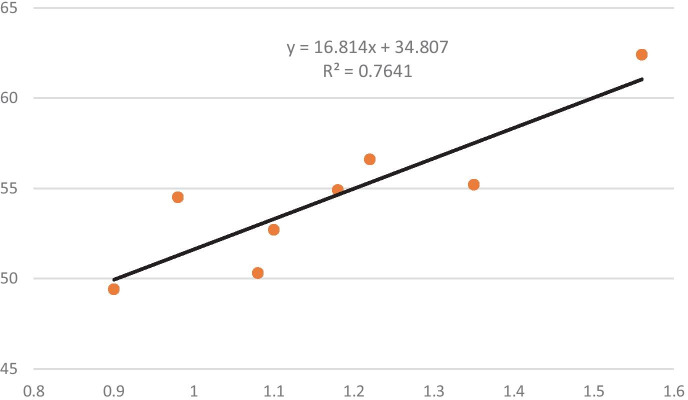
Relationship between WPI and WQI value

The Water Quality Index is most frequently used for assessing the suitability of water for drinking purposes. The value of this index is established by juxtaposing present water parameters with drinking water standards. Based on the assessment that has been carried out, some researchers have classified the quality of drinking water into five grades (Yadav et al., 2010; Akter et al., 2016; Loga et al., 2018). This grading provides water quality rating (WQR) with the following WQI: excellent water (0–25, grade A), good water (26–50, grade B), poor water (51–75, grade C), very poor water (76–100, grade D) and unsuitable for drinking (above 100, grade E). This list clearly shows that although water is deemed suitable for drinking, in some cases, it should not be consumed. It can be assumed that the water sources should be treated if the value of WQI was higher than 26. To check this, WQI, WPI and water clarity were compared (Table 7). The outcome of the assessment in this study points out to the relationship between WQI and WPI that is statistically significant (Fig. 2).

**Table 6:** The water at other stations belonging to A3 category

Parameter	Kock	Niewęgłosz	Buradów	Borki	Parczew	Białka	Rudka	Koczergi
SS	A1	A1	A1	A1	A1	A1	A1	A1
BOD	A1	A1	A1	A2	A1	A1	A1	A1
DO	A1	A1	A1	A1	A1	A1	A1	A2
TOC	A2	A3	A3	A3	A3	A3	A3	A3
pH	A1	A1	A1	A1	A1	A1	A1	A1
EC	A1	A1	A1	A1	A1	A1	A1	A1
SO_4_	A1	A1	A1	A1	A1	A1	A1	A1
Cl	A1	A1	A1	A1	A1	A1	A1	A1
NH_4_	A1	A1	A1	A1	A1	A1	A1	A1
KN	A2	A2	A2	A2	A2	A2	A2	A2
PO_4_	A1	A2	A2	A1	A1	A1	A2	A2
COD	A1	A2	A3	A2	A2	A2	A2	A3
Total	A2	A3	A3	A3	A3	A3	A3	A3

**Table 7:** Summary of WQI, WPI and water clarity values

Indicator	Kock	Niewęgłosz	Buradów	Borki	Parczew	Białka	Rudka	Koczergi
Clarity	A2	A3	A3	A3	A3	A3	A3	A3
WPI	0.90	1.10	1.18	1.56	1.08	0.98	1.22	1.35
WPI class	II	III	III	III	III	II	III	III
WQI	49.4	52.7	54.9	62.4	50.3	54.5	56.6	55.2
WQI gradient	B	C	C	C	C	C	C	C

According to the values of WQI and WPI, the grading of 7 stations out of 8 has the same classes. These classes are B and II, pure polluted and good water and C and III, moderately polluted and poor water. The value of WPI is easier to calculate than WQI. It is sufficient to calculate the mean values of the parameters of the first-class water quality standards in case of WPI (Gazette, 2016). Therefore, WPI could be used instead of WQI to assess drinking water suitability. However, due to the limited scope of analyses conducted in the paper, more extensive research should be carried out for this purpose.

High concentrations of nitrogen and organic carbon in the river basin are associated with the processes of mineralization and subsidence of peat bogs representing 40% of the surface. Bogs drainage causes emissions of CO_2_, CH_4_ and N_2_O. Along with global warming and C losses, this leads to reducing the variety of plant communities in peat ecosystems. The drop in water level contributes to releasing carbon and organic nitrogen into groundwater and afterwards into surface water (Berglund & Berglund, 2011; Dijkstra et al., 2012; Musarika et al., 2017; Korkiakoski et al., 2018). An increase in TOC and KN concentration is a direct cause of an increase in COD. Values of pollution indicators such as TOC and KN are part of the assessment of water pollution index (WPI) and the need for its treatment (clarity). However, no drinking water standards have been established for these indices. The analysis of studies described in this paper suggests that for organic soils, nitrogen and organic carbon can be important factors contributing to drinking water pollution (Brankov et al., 2012).

The values of chemical parameters of water could vary depending on the temporal and spatial situation. The growth in phosphate concentration is correlated with non-scheduled waste disposal, agricultural runoff and the use of pesticides and fertilizers that cause surface water pollution (Mirzaei et al., 2016; Sun et al., 2016; Grzywna & Bronowicka-Mielniczuk, 2020). It has been found in the examined water of the current study that the concentrations of arsenic, barium, boron, zinc, copper, aluminium, molybdenum and selenium were less than 0.01 mg dm^−3^. This was the reason for not including them in the analyses of drinking water quality (Gazette, 2017). Many factors directly and indirectly affect the physicochemical parameters and the level of water pollution. All physicochemical parameters of the water in the Tyśmienica River catchment meet the second class quality standards and have a good ecological status. Similar results were obtained for the Ganga river system, where the water met drinking water standards (Matta et al., 2017, 2020).

The assessment of drinking water quality is a fundamental requirement in the context of the emerging problems, where the availability of drinking water is threatened by natural factors and human activity. This study carried out in the Tyśmienica river basin was aimed to determine water pollution by using WPI and the mutual influence of chemical parameters. One of the possible outcomes of this study, drinking water indicators can be developed and improved. However, the use of the WPI to assess the suitability of water for drinking requires further detailed research. The results of the research can be used to improve water quality and the proper management of the river basin in the future.

## Conclusions

In the current study, the values of all physico-chemical parameters are consistent with the values of such elements under undisturbed conditions, which contribute to compliance with the requirements of the Water Framework Directive. Surface waters at the Tyśmienica river basin are of the second quality class. The ecological status of waters of the Tyśmienica basin in 2017 was good. The general assessment of water quality using the WPI revealed statistically significant differences between checkpoints. The WPI assessment, as a function of twelve physical and chemical parameters of water quality, showed that surface water clarity based on the calculated 1.2 was characterized as moderately contaminated, which corresponds to water purity class III. However, at two stations, namely Kock and Białka, water was characterized as pure, which corresponds to purity class II. River water has met the requirements as a source water used for human consumption (clarity category A1 and A2). Due to high concentration of TOC and COD (category A3), high efficiency treatment processes should be used to ensure that water is suitable for drinking. For certain parameters, it will be sufficient to use typical physical treatment processes. The WPI water quality classification is similar to the results of the ecological status assessment and it can be used to determine the overall suitability of water for drinking. The alignment of national water legislation with the WFD and the introduction of appropriate management of water resources have contributed to achieving the EU’s objectives. The results of the current study can form the basis for further monitoring of environmental changes in this aquatic ecosystem. ANOVA analysis showed that the values of the most specific chemical parameters did not differ significantly. Only ammonia and COD showed significant differences among the stations.

## Data Availability

All data generated or analyzed during this study are included in this published article (and its supplementary information files).
